# Criticisms

**Published:** 1873-02

**Authors:** W. E. Driscoll


### Criticisms.

BY W. E. DRISCOLL.

That a large portion of the contributions to the dental journals are written without proper forethought and preparation, is generally admitted and deprecated. That a part of this class of writing calls for criticism and refutation for the credit of the profession, is also admitted. That the performance of this duty of criticism is an unpleaaant task, all know who have tried it.

That the editors of the dental papers can not, and must not be depended upon to do all this work, is evident from various reasons. They can not always afford to make the enemies that seem to be an unavoidable result sometimes of the fairest review of another's writing. It would in one case cripple the journal, or make enemies for the college that it was connected with, or injure the business of the firm that published.

If the party criticised would always confine himself to the subject or principle in question, there need be nothing unpleasant about such discussions. But if his case seems in jeopardy, he often takes the course the lawyer advised his student to adopt. That if the facts in any case in court should proove to be against his client, he must attack the opposing lawyer. Thus diverting attention from the badness of his case if possible. But such a course will not always win, and especially is it likely to fail in a discussion before a learned profession.

Where there is obviously no motive for a personal contest, one does not always see the necessity of a disclaimer to every sentence bearing upon the subject in hand. And experience has shown that it does not in every instance better matters to claim innocence of ill intent.

In view of these facts, I have concluded that whenever I encounter an article in our periodicals advocating a principle or process that I believe, or know to be erroneous, or ill advised, and that it will not be controverted or corrected without I do so, I shall perform the duty to the best of my ability, whether my conclusions are flattering to the criticised or not. Yet, always, I trust, in terms that will bear upon their face evidence of no unkindness toward any individual. And should my motives be misconstrued as they have been, I shall not condescend to notice any such attacks, believing that I would forfeit the respect of all right minded readers if I did.

